# Towards high-integrity biodiversity credits: balancing commensurability, ecological complexity and governance

**DOI:** 10.1098/rspb.2025.0990

**Published:** 2025-08-20

**Authors:** Eun Hye Kim, Adrian Dellecker, Richard Field, P. J. Stephenson, Franziska Schrodt

**Affiliations:** ^1^ School of Geography, University of Nottingham, Nottingham NG7 2RD, UK; ^2^ Center for Sustainable and Inclusive Business, International Institute for Management Development, CH-1001 Lausanne, Switzerland; ^3^ Laboratory for Conservation Biology, Department of Ecology & Evolution, University of Lausanne, CH-1015 Lausanne, Switzerland; ^4^ SSC Species Monitoring Specialist Group, IUCN, 1196 Gland, Switzerland

**Keywords:** biodiversity finance, nature market, biodiversity credit integrity, biodiversity data standard

As the rapid decline in biodiversity exacerbates climate risks and threatens human wellbeing, biodiversity credits are emerging as a promising yet challenging mechanism for mobilizing conservation finance. Some scepticism stems from disappointment in the voluntary carbon market, which has unresolved challenges related to verification, additionality and social safeguards [1]. Some critics contend that biodiversity credits risk redundancy with existing market instruments, including nature-based carbon credits or payments for ecosystem services. Moreover, the current biodiversity metrics landscape is fragmented, creating barriers for end-users attempting to navigate the emerging nature credit markets. Against this backdrop, the development of biodiversity credits might seem premature or ill-advised to many observers.

In this context, Wauchope *et al.* [2] pose a critical question—‘What is a unit of nature?’—implicitly raising the fundamental question whether we have been focusing on the right unit of measurement in our quest to protect and restore nature, particularly amidst proliferating nature-based solutions. Any such unit must be both quantifiably expressible (commensurable) and ecologically meaningful (reflecting the state of nature). However, achieving both in a single metric is challenging owing to inherent trade-offs. While a more pluralistic approach is considered, we discuss essential additional requirements for making progress on genuine nature-positive outcomes. Confronting the inherent complexity of nature’s measurement demands, the discussion extends beyond scientific discourse and calls for coordinated accountability across sectors and the implementation of the safeguards. For this, we evaluated 11 biodiversity credit (or certificate) suppliers’ methodologies and governance frameworks against six essential elements and detailed sub-criteria from the International Advisory Panel on Biodiversity Credits’ (IAPB) ‘Design criteria for high integrity measurement of biodiversity credits’ ([3], see the electronic supplementary material for detailed methods). We emphasize the importance of robust policies, transparent practices based on standard protocols and equitable social engagement, highlighting the gaps in the current and desired state of high-integrity market.

## 1. Biodiversity credits should not be trapped in the carbon credit framework

The current dissonance over a single unit of measurement stems primarily from an approach to biodiversity that is largely fixated on the carbon credit framework. Carbon credits utilize a standardized unit of measurement—tonnes of CO₂ equivalent (CO₂e)—which functions as a commensurable unit across geographical regions, sectors and value chains. This uniformity is enabled by reducing the inputs (greenhouse gases from multiple sources) to a single, uniform pathway (the greenhouse effect); it has facilitated commodification of emission reductions, allowing global trade and alignment with existing accounting frameworks. There are calls for a similar approach to biodiversity metrics, driven by market-based conservation and corporate sustainability reporting needs. However, as Wauchope *et al.* [2] find, these efforts face ‘monumental challenges’ (also see [4]), not only technical but also epistemological, highlighting the tension between standardizing biodiversity metrics and sufficiently incorporating ecological complexity.

Standardization for market integration typically requires abstraction, which first involves identifying and demarcating measurable ecological objects (e.g. species, habitats or ecosystem functions), isolating them from their ecological context and then reducing these context-stripped objects into numerical values. This process aims to produce site-agnostic units to facilitate trade. Yet, such reductionist approach over-simplifies our view of biodiversity, severing crucial interactions and interdependence among and between organisms and their environmental contexts. We also risk losing sight of biodiversity’s intrinsic values—that is, inherent value independent of utility to humans—thereby prioritizing market utility over ecological integrity and meaning [5].

The case of carbon credits illustrates another important risk associated with simplified standardization: the inevitable presence of 'deep uncertainty', which Wells *et al.* [6] defined as ‘unknown unknowns’, the inherent unpredictability of biotic carbon change that is rooted in the disintegration of complex socioecological system dynamics. This results in discrepancies between anticipated (forecast) and actual carbon outcomes, which has cascaded into a series of integrity issues with carbon credits, starkly evident in reforestation projects [7]. Given the generally greater uncertainties associated with biodiversity than carbon, this issue is of even more profound concern for biodiversity credit markets. While standardization is critical for scaling biodiversity finance, trying to create fungible units is likely to undermine the ecological integrity it aims to protect. Biodiversity is multidimensional, encompassing genetic, species and ecosystem diversity across multi-spatial and temporal scales [8], and has multiple types of value to people and corporates. On the other hand, any measurement system that seeks to fully encapsulate these complexities risks becoming excessively descriptive as well as too costly, prioritizing ecological specificity without offering clear applicability of feasibility for a credit market. Recognition of these problems causes us to think outside the carbon framework.

## 2. Protocols first: where standardization really matters

The increasing recognition of biodiversity’s ecological, economic and cultural significance has drawn a diverse array of actors into conservation efforts, ranging from governments and multinational corporations to Indigenous communities and international NGOs. Each of these stakeholders operates with distinct priorities, resource constraints and institutional frameworks, requiring metrics tailored to their specific objectives and decision-making contexts [4]. Moreover, various emerging financial mechanisms for biodiversity—such as biodiversity credits, which encompass multiple credit types aligned with different levels of mitigation hierarchy—require tailored metrics and methodologies for different nature conservation goals and objectives.

The inherent challenges with standardization and divergent market demands underscore a broader principle that has gained increasing support from both scholars and practitioners, including Wauchope *et al.* [2]: *different metrics are necessary for different purposes* [2,4]. While this pluralistic approach is relatively flexible to reflect the complexity of nature, it also exacerbates the proliferation of diverse and sometimes incompatible metrics, introducing unresolved risks inherent to abstraction and standardization. Further complicating the landscape is the rapid adoption of market-driven technological solutions. While academic research has increasingly incorporated new tools such as remote sensing and atrificai intelligence (AI)-driven monitoring, market-driven solutions operate at a markedly different pace, prioritizing scalability over methodological rigour, ecological validity and cross-validation with empirical field data. The divergence between these two knowledge systems can lead to both productive synergies and critical disconnects.

In this context, resolving such increasing complexity through a few selected sets of standardized metrics is likely to be counterproductive, potentially leading to more disputes. Instead, efforts should focus on harmonizing the end-to-end data protocols to standardize across the entire life cycle—from data collection through integration to distribution processes—rather than final metrics themselves. Such protocols would provide the necessary flexibility to accommodate diverse data repositories sourced from multiple collection methods across varying scales, while ensuring these datasets remain harmonized for meaningful comparative evaluations to inform policies and investment decisions. This approach offers the additional advantage of allowing key metrics to evolve and become increasingly refined as datasets accumulate over time. Enabling the aggregation of data over long-term monitoring would be particularly valuable to develop predictive models. Indeed, large-scale initiatives, including Biodiversa+ and EuropaBON, have already begun proposing strategies for an optimal way to harmonize data protocols at the European Union level [9].

## 3. High-integrity metrics: addressing systemic gaps in biodiversity credit market readiness

The relevance of such harmonized data streams, however, hinges entirely on their integrity. This shifts the discussion beyond scientific frameworks alone, toward high-integrity governance principles collectively upheld by a broad spectrum of stakeholders, both within and beyond market systems. While scientific rigour remains critical for understanding ecological processes and quantifying change, accountability must be distributed across actors to enable cross-sectoral monitoring, verification and enforcement. Recognizing this need, the World Economic Forum (WEF) released its first set of high-level governance and integrity principles at the 15th Conference of the Parties to the Convention on Biological Diversity (COP15), which were further detailed at COP16 by the IAPB [1,3] and the Biodiversity Credit Alliance (BCA). Currently, the BCA, IAPB and WEF are collaborating to update these high-level principles based on lessons learned from the operation of biodiversity credit pilot markets [10].

**Figure 1.**
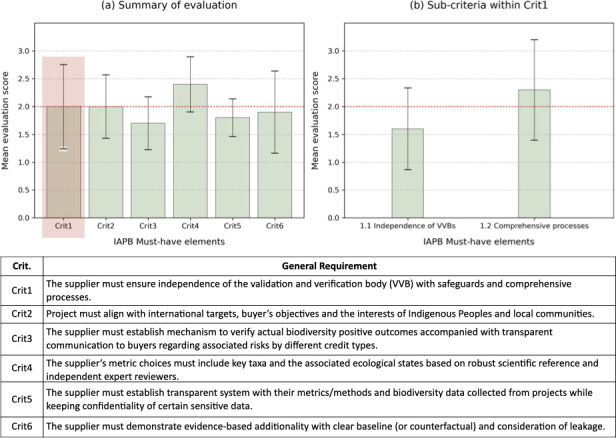
A summary of evaluation results for 11 biodiversity credit (or certificate) suppliers. A score of 0 for a given criterion indicates that none of the IAPB sub-requirements was met, while 1 (minimal alignment), 2 (partial alignment) and 3 (full alignment) indicate progressively higher levels of fulfilment. The horizontal dotted red line indicates the mean value across all six criteria. Chart (a) shows scores for all six IAPB ‘must have’ criteria that are listed in the table below; (b) divides criterion 1 into its two sub-criteria. Error bars show the standard deviation of scores across the suppliers. See the electronic supplementary materials for full, detailed evaluation results across credit suppliers.

Across these initiatives, core integrity requirements have remained consistent: independent third-party verification, transparency throughout project design and implementation, and inclusive social equity mechanisms. The IAPB’s design criteria formalize these priorities through six essential criteria in the application of metrics and measurements (figure 1). Each of these six essential criteria is then subdivided into specific sub-elements that outline the detailed requirements. We applied these detailed sub-requirements as an evaluation framework to assess 11 biodiversity credit suppliers' documented policies and methodological approaches, determining the extent to which they align with these criteria. Each sub-requirement fulfilment was converted to a numerical value between 0 (no evidence) and 1 (clear evidence). These values were then averaged to assign a final score on a 4-point scale from 0 (no alignment) to 3 (full alignment) for each parent criterion (see the electronic supplementary material for detailed methods).

Our results indicate that the market is still in the developing stage, with an overall mean of 2, while Criterion 3, verifiable positive outcomes and transparent disclosures of risks, demonstrates the least progress (score 1.7, figure 1a). Most suppliers present well documented methodologies, supported by scientific references and expert reviews. This is evident in Criterion 4 (science-based verifiable measurements) receiving the highest average score (2.4). Similarly, many suppliers have well documented validation and verification processes (score of 2.3 for Crit1.2, figure 1b). However, a critical gap exists in ensuring independence of validation and verification bodies (VVBs), leading to the lowest score (1.6) for Crit1.1. A total of 8 out of 11 suppliers did not engage third-party accredited VVBs, and conflict-of-interest safeguards are often absent or insufficient. VVBs are rarely rotated, and audit criteria lack transparency, raising concerns about the consistency and impartiality of oversight. In addition, most suppliers did not clearly define credit types with linked payment schedules tied to verification or disclose associated risks (score of 1.7 for Crit3). These results highlight a disconnect between documented procedures and how they are executed in practice. Given the substantial financial ties between credit suppliers and VVBs, the current practice may raise reasonable integrity concerns. This is because most credit suppliers select and fund their own VVBs for compliance with their ‘internal’ standards. This concern also relates directly to Criterion 3, which addresses mechanisms penalizing non-compliance and transparent disclosure of associated risks to buyers. While credit suppliers benefit financially from ‘levies’ on credit issuance, such concentration of control, including rule-setting, testing and self-imposed penalties, raises concerns about impartiality, particularly in markets that rely on buyer trust and environmental integrity. Similar concerns on perverse incentives in carbon credits have been consistently raised, with calls for a redistribution of controls, for example, by revoking delegation of decisions about carbon benefit claims from credit suppliers to standard and independent accreditation bodies [11].

Social equity also lags; while engagement with Indigenous Peoples and local communities is widely acknowledged, only about half have established comprehensive FPIC (free, prior and informed consent) protocols, and none of the suppliers explicitly indicates that its data usage complies with CARE (collective benefit, authority to control, responsibility, ethics) principles or comparable policies. Integrating the CARE principles to conservation activities recognizes Indigenous data sovereignty and self-determination, promotes data governance that reflects Indigenous values, and ensures data practices produce benefits for Indigenous communities. Indeed, the data generated from Indigenous territories can be more valuable than the credits themselves, as these data inform critical conservation decisions once digitized and enriched with Indigenous knowledge. It is therefore essential that Indigenous communities retain control over how their territorial data are used, and are able to benefit from the ongoing value of their data, not just the initial economic gains from the issuance of credits. The absence of a data-sharing policy with Indigenous Peoples may imply only partial acknowledgement of their roles, perceived more as beneficiaries than as legitimate decision makers and knowledge holders.

Without safeguards to address these operational and governance risks, advances in metrics alone will not prevent the replication of failures seen in carbon markets, such as over-crediting, greenwashing and inequitable benefit sharing. The technical challenges highlighted by Wauchope *et al.* [2], such as leakage, permanence and additionality verification, are all originally drawn from the mistakes and lessons learned from the carbon market. While these are all important and relevant issues, the areas of substantial under-development identified in our analysis, including verification independence, transparency in risk, and data governance and equity sharing, would only amplify these technical limitations if not properly addressed. The ongoing demand for high-integrity biodiversity credits from suppliers, buyers, civil society and Indigenous Peoples, therefore, is not merely about being ethically correct; it actively drives technical improvement by encouraging the integration of structural accountability mechanisms that uphold, rather than simplify, the inherent complexity and integrity of nature.

## 4. Operationalizing integrity: the critical roles of transparency based on harmonized protocols and regulatory oversight

**Figure 2.**
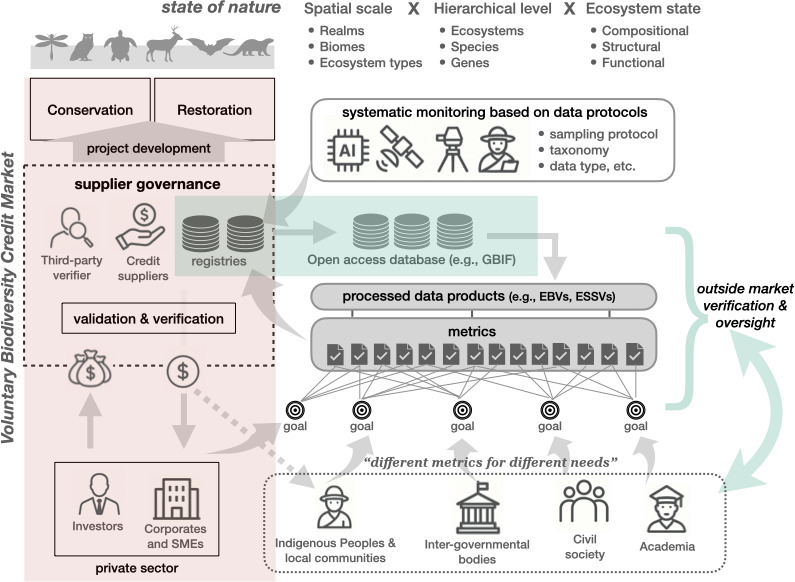
An illustration of the broad context of voluntary biodiversity credit markets. They are highly vertically integrated (red shading), inherently prone to operate within a closed ecosystem. This necessitates greater transparency and disclosure requirements, through appropriate social monitoring and regulatory interventions. Shared accessibility to biodiversity data and registries (blue shading) and standard protocols are key routes to reduce information asymmetries and to genuinely enhance integrity standards for nature-positive outcomes. AI, artificial intelligence; GBIF, Global Biodiversity Information Facility; EBVs, Essential Biodiversity Variables; ESSVs, Ecosystem Services Variables; SMEs, Small and Medium Enterprises.

To build trust and navigate metric complexity, operationalizing integrity in biodiversity credit markets requires two foundational enablers: systemic transparency and regulatory intervention. Transparency across the entire credit lifecycle, including harmonized biodiversity data based on standardized protocols, enables self-regulation and mutual accountability (figure 2). In parallel, robust government policies and regulatory frameworks are necessary to ensure that such transparency is maintained, enforced and aligned with public interest.

The nascent biodiversity credit market is exposed to multiple market failures that demand market regulatory interventions [12]. These include information asymmetries between buyers and sellers, creating adverse selection risks, unpriced externalities affecting ecological outcomes, and weak competitive pressures, leading to potential market manipulation. Yet, recent cases illustrate the difficulty of implementing regulatory interventions. In the UK, mandatory Biodiversity Net Gain (BNG) has delivered less than 13% of projected habitat targets, hampered by limited monitoring resources and regulatory loopholes [13]. Similarly, the European Commission reversed course on its 2023 mandatory European Sustainability Reporting Standards (ESRS) under pressure from industry [14]. This has been strongly criticized on various grounds, including that uncertainty hinders investment, that regulatory reporting is important for both nature and businesses, and that scientific and transparency standards should be stronger, not weaker [15]. Poorly enforced or diluted regulation risks legitimizing greenwashing. To counter this, transparency must be embedded within regulatory frameworks, not just as a procedural measure but as a substantive mechanism for cross-sectoral accountability. Biodiversity credits operate within a vertical market structure where information asymmetries can emerge at multiple levels: not only between buyers and sellers but also between market participants and external stakeholders (figure 2). Our analysis reveals that validation and verification processes are predominantly governed by suppliers, with restricted registry access and limited public disclosure. Such practices compromise verification and perpetuate information asymmetries.

Structured, accessible disclosure of measurement methodologies and outcomes is required. While sensitive data require controls on release, greater openness fosters collective learning and allows external stakeholders to independently verify claims. As discussed, standardizing essential data protocols is critical in underpinning transparent data practices. Without standardizing protocols, fragmented data from multiple sources and inconsistent sampling approaches across varying scales may create greater confusion in the market, amplifying information asymmetries even under transparent data policies. This, in turn, undermines the effectiveness of well designed policy frameworks, leaving the market susceptible to manipulation.

Efforts to integrate biodiversity into market-based systems are still in their infancy, and the foundational components—methodological development, governance integrity, transparency and regulatory oversight—must be advanced concurrently, not sequentially. While the challenges are substantial, the trajectory of the voluntary carbon market offers both a cautionary tale and an instructive precedent. Despite persistent criticism and operational pitfalls, the carbon credit market demonstrated a notable growth from 2021 to 2023, signalling substantial latent demand within private sector sustainability financing.

Currently, there is a narrow but critical policy window. The inflow of capital represents an opportunity to embed high-integrity governance early on in the development of the biodiversity credit market. However, doing so requires moving beyond incrementalism and technocratic trial and error. The task ahead is not simply refinement of monitoring, reporting and verification processes but systemic coordination of the key actors, to ensure that this new market instrument delivers measurable, equitable and durable biodiversity outcomes.

## Data Availability

All datasets are available in the online electronic supplementary material [16].
